# Combining Metabolomics and Transcriptomics to Reveal the Regulatory Mechanism of Taproot Enlargement in *Panax ginseng*

**DOI:** 10.3390/ijms24065590

**Published:** 2023-03-15

**Authors:** Meng Zhang, Yingxin Sun, Ping Di, Mei Han, Limin Yang

**Affiliations:** Co-Constructing Key Laboratory by Province and the Ministry of Science and Technology of Ecological Restoration and Ecosystem Management, College of Chinese Medicinal Material, Jilin Agricultural University, Changchun 130118, China; zhangmeng@mails.jlau.edu.cn (M.Z.);

**Keywords:** garden ginseng, taproot enlargement, glycometabolism, plant hormone lignin

## Abstract

Ginseng is regarded as the “king of herbs” in China, with its roots and rhizomes used as medicine, and it has a high medicinal value. In order to meet the market demand, the artificial cultivation of ginseng emerged, but different growth environments significantly affect the root morphology of garden ginseng. In this study, we used ginseng cultivated in deforested land (CF-CG) and ginseng cultivated in farmland (F-CG) as experimental materials. These two phenotypes were explored at the transcriptomic and metabolomic levels so as to understand the regulatory mechanism of taproot enlargement in garden ginseng. The results show that, compared with those of F-CG, the thickness of the main roots in CF-CG was increased by 70.5%, and the fresh weight of the taproots was increased by 305.4%. Sucrose, fructose and ginsenoside were significantly accumulated in CF-CG. During the enlargement of the taproots of CF-CG, genes related to starch and sucrose metabolism were significantly up-regulated, while genes related to lignin biosynthesis were significantly down-regulated. Auxin, gibberellin and abscisic acid synergistically regulated the enlargement of the taproots of the garden ginseng. In addition, as a sugar signaling molecule, T6P might act on the auxin synthesis gene *ALDH2* to promote the synthesis of auxin and, thus, participate in the growth and development of garden ginseng roots. In summary, our study is conducive to clarifying the molecular regulation mechanism of taproot enlargement in garden ginseng, and it provides new insights for the further exploration of the morphogenesis of ginseng roots.

## 1. Introduction

*Panax ginseng* C. A. Meyer is a medicinal herb, used in the forms of dried roots and rhizomes, and has been used as a Chinese herbal medicine for thousands of years in China and in various other countries [1]. According to the theory of traditional Chinese medicine, ginseng has the functions of invigorating vitality, recovering pulse, nourishing the spleen and benefiting the lungs, promoting body fluid production and nourishing blood, tranquilizing the mind and improving intelligence, etc. It has an extremely high medicinal value [2]. Modern pharmacological studies have also proven that ginseng has various pharmacological effects, such as immunity-enhancing [3], anti-aging [4], anti-fatigue [5] and anti-tumor effects [6]. In China, due to excessive foraging and the lack of wild ginseng resources, in order to meet the market demand, people began to study the artificial cultivation of ginseng, commonly known as garden ginseng [7,8]. There are two main ways to cultivate garden ginseng: one way is to cultivate ginseng on land after deforestation, which we call “cutting forest ginseng”, and the other is to cultivate ginseng on farmland, which we call “farmland ginseng”. Because the soil in deforested areas is fertile and has sufficient nutrients, the taproots of cutting forest ginseng are more enlarged, which makes the yield higher [9,10].

The enlargement of roots is a complex biological process involving morphogenesis and dry matter accumulation, which are jointly regulated by genetic, environmental and physiological factors [11,12]. The thickening mechanism of taproots has been extensively studied in anatomy and physiology in recent decades, and it has been found that plant hormones are signal molecules that naturally exist in plants, affect the growth and development of plants and play very important roles in the process of plant root enlargement [13]. Studies have shown that the contents of auxin (IAA) increase rapidly in the early stages of storage root expansion, so it might play important roles in promoting the initial thickening of storage roots [14]. The contents of abscisic acid (ABA) [15] and jasmonic acid (JA) [16] have been found to increase significantly during the process of root or rhizome swelling. However, the effects of cytokinin (CTK) and gibberellin (GA) on the enlargement of roots or rhizomes are not consistent. In previous studies, the content of CTK increased significantly during the development of sweet potato storage roots, but it decreased significantly during the expansion of potato tubers [17,18]; when exogenous GA was applied, it affected cell division and lignin synthesis, thereby inhibiting root growth, but it could promote the secondary growth of ginseng roots by improving the development of storage parenchyma cells [19,20,21]. These results also indicate that the regulation of plant hormones in plant growth varies with plant species. Additionally, secondary metabolites can also regulate plant phenotypes. Ginsenosides, the medicinal components of ginseng, can regulate the formation of adventitious roots in ginseng through the new *PgCLE45–PgWOX11* regulation module [22].

Transcriptomics is a molecular approach to studying the expressions of genes at the RNA level under specific physiological conditions, helping to identify differentially expressed genes and understand phenotypic differences [23]. In sweet potato, it has been verified that, in the early stages of storage root formation, lignin biosynthesis genes are down-regulated and starch and sucrose biosynthesis genes are up-regulated [24]. It has been found that the gene *SRD1* can affect the formation and development of storage roots by regulating auxin synthesis [25]. In addition, some transcription factors, such as *WRKY, bHLH, NAC* and *bZIP*, have been found to play important roles in plant growth and development, physiological metabolism and signal transduction [26,27,28]. However, the discovery of key genes related to taproot enlargement in ginseng is still limited.

Ginseng is a slow-growing plant with a cultivation period of up to 4–6 years; therefore, the enlarged growth of its roots is particularly important for its productivity [19]. However, the research on ginseng has mainly focused on its pharmacological effects on the human body, and in-depth research on its physiological characteristics has rarely been conducted. Therefore, in this study, a combination of metabolomic and transcriptomic techniques was used to analyze the taproots of cutting forest ginseng (CF-CG) and farmland ginseng (F-CG), which had significant differences in the degree of taproot enlargement, in order to identify the differential metabolites and the related differentially expressed genes. These analyses indicated that the biosynthesis of carbohydrates, plant hormones, lignin and ginsenoside, as well as related genes, was involved in the regulation of taproot enlargement in ginseng, providing new insights into the molecular regulation of taproot enlargement in garden ginseng.

## 2. Results

### 2.1. Growth and Development Indicators and Sugar Content

**Figure 1:**
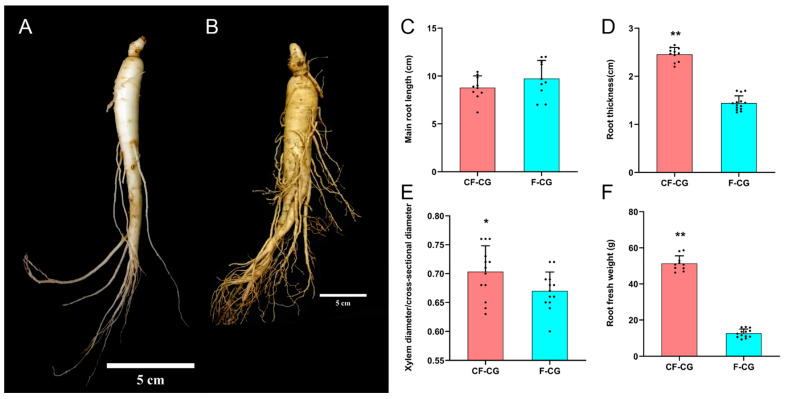
Growth and development of taproots of garden ginseng in different regions. (**A**): Garden ginseng cultivated in farmland (F-CG); (**B**): garden ginseng cultivated in deforested land (CF-CG), with the linear scale = 5 cm. (**C**): main root length; (**D**): main root thickness; (**E**): ratio of xylem diameter to cross-sectional diameter; (**F**): fresh weight of root (points in the figure represent individual values, and error bars represent standard errors; *n* = 12 (* *p* < 0.05; ** *p* > 0.01; the significance of the difference was analyzed using *t*-test).

**Figure 2:**
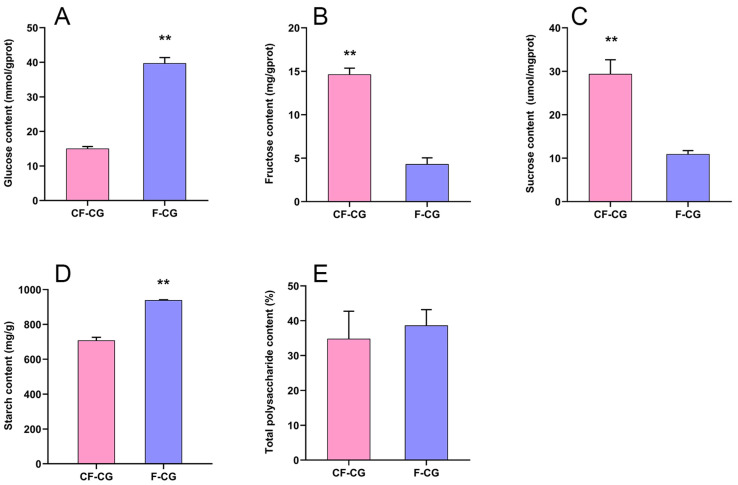
Determination of carbohydrate content in main roots of F-CG and CF-CG. (**A**): glucose; (**B**): fructose; (**C**): sucrose; (**D**): starch; (**E**): total polysaccharides (** *p* < 0.01; the significance of difference was analyzed using *t*-test; vertical bars indicate the mean value ± standard deviation from three independent experiments).

The root characteristics of the cultivated garden ginseng were significantly different due to the difference in growth environments. We studied the important developmental indicators of the roots of CF-CG and F-CG (Figure 1A,B). The results show that, compared with F-CG, the lengths of the main roots of CF-CG were not significantly different (Figure 1C), accounting for about one-half to one-third of the total root length. The roots’ thicknesses were increased by 70.5% (Figure 1D), and the proportion of the xylem diameter of the taproots to the cross-sectional diameter was significantly increased (Figure 1E), indicating that the growth environment in the deforested area could significantly promote the enlargement of the taproots of ginseng. At the same time, the enlargement of the taproots of CF-CG resulted in a significant increase in the roots’ fresh weight, which was significantly increased by 305.4% compared with that of F-CG in the same period (Figure 1F), greatly improving the yield of garden ginseng. Carbohydrates are the main product of photosynthesis, and provide energy for the growth and development of ginseng. We measured the contents of glucose, fructose, sucrose, starch and total polysaccharides in the taproots of the ginseng. The results show that, compared with F-CG, the contents of fructose and sucrose in CF-CG were increased by 2.38 times and 1.68 times, respectively (Figure 2B,C). In contrast, the contents of glucose and starch were lower; that is, they had decreased by 1.24 times and 0.32 times, respectively (Figure 2A,D). There was no significant difference in the total polysaccharide contents accumulated between CF-CG and F-CG (Figure 2E).

### 2.2. Ginsenoside Content

**Figure 3:**
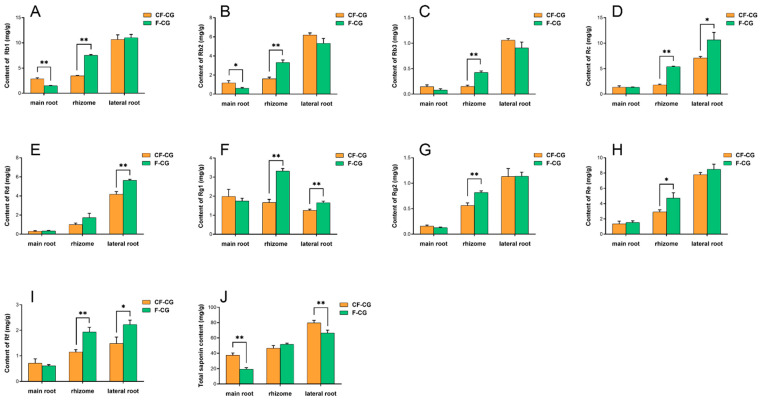
Determination of ginsenoside components in main roots of F-CG and CF-CG. (**A**): ginsenoside Rb1; (**B**): ginsenoside Rb2; (**C**): ginsenoside Rb3; (**D**): ginsenoside Rc; (**E**): ginsenoside Rd; (**F**): ginsenoside Rg1; **G**: ginsenoside Rg2; (**H**): ginsenoside Re; (**I**): ginsenoside Rf; (**J**): determination of total ginsenoside, etc. (* *p* < 0.05; ** *p* > 0.01; the significance of the difference was analyzed using *t*-test; vertical bars indicate the mean value ± standard deviation from three independent experiments).

The dried roots and rhizomes of ginseng are used for medicinal purposes, and ginsenosides are the main medicinal components of ginseng. We analyzed the ginsenoside components in the main roots, rhizomes and lateral roots of three parts of CF-CG and F-CG using HPLC (Figure 3A–J). The results show that the contents of the other nine monomer saponins and total saponins were distributed in the order of the main root < rhizome < lateral root, except for ginsenoside Rg1, which was higher in the main root (Figure 3F). In the main roots of the ginseng, the contents of the nine monomeric saponins and total saponins of CF-CG were higher than those in F-CG, with significant differences in the total contents of ginsenosides Rb1 (Figure 3A) and Rb2 (Figure 3B) and total saponins (Figure 3J), reaching 2.83 mg/g, 1.17 mg/g and 37.39 mg/g, respectively. The content of total saponins in the lateral roots was also significantly higher than that in F-CG, whereas the contents of the nine monomeric saponins and total saponins in the rhizomes showed the opposite trend. Interestingly, the main root of the garden ginseng accounted for the largest proportion of the whole garden ginseng. The enlargement of the main roots of CF-CG might be related to the increase in the secondary metabolized ginsenoside content.

### 2.3. Metabolome Data Analysis

**Figure 4:**
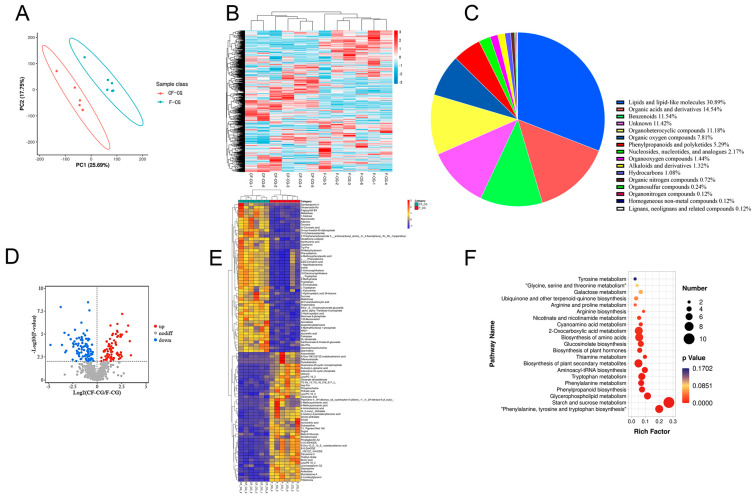
Basic metabolite information of F-CG and CF-CG based on non-targeted metabolomics. (**A**): Principal component analysis of the metabolites identified in F-CG and CF-CG. (**B**): Clustering analysis of F-CG and CF-CG samples. (**C**): A pie chart depicting the class of metabolites identified. (**D**): Volcano map showing differentially accumulated metabolites (DAMs) up-regulated, down-regulated or unchanged between F-CG and CF-CG samples. (**E**): Differential expression of DAMS between F-CG and CF-CG. (**F**): KEGG enrichment analysis of DAMs between F-CG and CF-CG.

In order to more comprehensively explore the regulation of taproot enlargement in garden ginseng, a non-targeted UPLC-MS analysis was used to identify the metabolites in the CF-CG and F-CG samples. A total of 832 metabolites were identified, which could be divided into more than 15 categories. Lipids and lipid molecules (30.89%) were the main metabolites (Figure 4C). A principal component analysis (PCA) of the CF-CG and F-CG samples showed that the two groups of samples could be effectively separated (Figure 4A). A cluster analysis of the samples also showed that the biological repeatability within the sample group was good (Figure 4B). The different samples could be clearly distinguished, indicating that the metabolome data were highly reliable.

In order to identify the differentially expressed metabolites (DEMs), we used a ratio of >=2 or a ratio of <=1/2, q-value of <=0.05 and VIP ≥ 1 as the standard. A total of 116 differential metabolites were identified, and the volcano plot shows that 55 metabolites were down-regulated and that 61 metabolites were up-regulated (Figure 4D). Using a heatmap analysis, we observed significant differences in the abundance of primary metabolites between the different phenotypes (Figure 4E). Compared with F-CG, the main metabolites that significantly accumulated in CF-CG with swollen taproots were mostly sugars and amino acids, such as sucrose, trehalose, maltose, tryptophan and tyrosine, while some organic acids and their derivatives, phenylpropanoid and polyketide compounds, lipids and other compounds were significantly accumulated in F-CG. The differential metabolites were further enriched using the KEGG pathway analysis (Figure 4F). The results show that they were mainly enriched in the pathways of starch and sucrose metabolism; phenylalanine, tyrosine and tryptophan biosynthesis; and plant secondary metabolite biosynthesis. They were also significantly enriched in phenylpropane biosynthesis and plant hormone biosynthesis. Therefore, we speculate that carbohydrate metabolism and the biosynthesis of different metabolites, such as amino acids, phenylpropane, plant hormones and secondary metabolites, are involved in the regulation of CF-CG taproot enlargement.

### 2.4. Transcriptome Data Analysis

**Figure 5:**
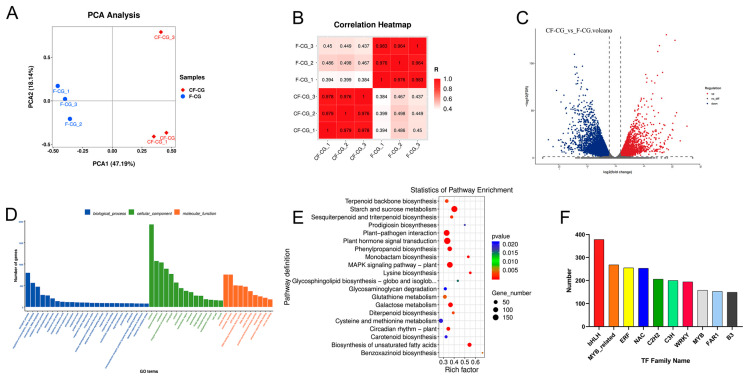
Basic genetic information of F-CG and CF-CG based on transcriptomics. (**A**): Principal component analysis of genes identified in F-CG and CF-CG. (**B**): Correlation analysis of F-CG and CF-CG samples. (Color indicates the level of correlation for each sample, from low (pink) to high (red), and Z score indicates the correlation coefficient between every two samples.) (**C**): Volcano plot showing differentially accumulated genes (DAGs) up-regulated, down-regulated or unchanged between F-CG and CF-CG samples. (**D**): GO enrichment analysis of DAGs for F-CG and CF-CG samples. (**E**): KEGG enrichment analysis of DAGs for F-CG and CF-CG samples. (**F**): A family of differentially expressed transcription factors in F-CG and CF-CG.

We studied the changes in gene expression levels between cutting forest ginseng (CF-CG) and farmland ginseng (F-CG). Six cDNA libraries were constructed, and RNA sequencing was conducted using these two samples as the sources of total RNA (Table 1). A total of 321,085,106 raw reads were generated, and 315,230,224 clean reads were obtained by removing low-quality reads. After de novo assembly, mapping to ensembles and redundancy removal, 65,913 unigenes were obtained with a total N50 length of 1476 nucleotides (Table 2). Therefore, the experimental data obtained via sequencing were of high quality and met the conditions for subsequent experimental analyses. Of the 65,913 unique sequences, the following were annotated in the GO, KEGG, Pfam, SwissProt, eggNOG and NR databases using BLASTX: 45.69%, 35.05%, 39.05%, 37.36%, 50.82% and 51.80%, respectively (Table 3). The principal component analysis and correlation analysis of the samples showed that the biological repeatability within the sample group was good and the samples could be grouped together (Figure 5A,B). Different samples could be clearly distinguished and had different correlations.

In order to explore the candidate genes that caused differences in the enlargement of the taproots of garden ginseng, DEGs were selected based on log2 (fold change) of >1 (up-regulated) or <1 (down-regulated). Compared with F-CG, a total of 9960 DEGs were identified in CF-CG, and a volcano plot was constructed for a further analysis, showing that 4409 DEGs were up-regulated and 5551 DEGs were down-regulated (Figure 5C). Furthermore, the GO functional enrichment analysis of the 9960 DEGs revealed their involvement in biological processes (i.e., the biological process, the regulation of transcription, DNA-templated transcription and the DNA-templated defense response), cellular components (i.e., the nucleus, plasma membrane and cytoplasm) and molecular functions (i.e., protein binding, molecular function and ATP binding) (Figure 5D). For the further identification of the metabolic pathways of differential gene enrichment in taproot enlargement in garden ginseng, the 9960 DEGs were mapped to the KEGG database, and the top 20 metabolic pathways with the smallest *p* value were enriched, as shown in Figure 5E; these were related to plant hormone signal transduction, starch and sucrose metabolism, plant–pathogen interaction, MAPK signaling pathway-plant, galactose metabolism and phenylpropane biosynthesis, and they were highly correlated. Therefore, this study mainly focuses on the metabolic pathways of plant hormones, phenylpropane compounds and carbohydrates.

Transcription factors are proteins that can bind to specific DNA sequences to initiate and regulate gene expression by recognizing and binding cis-acting elements in gene promoter regions. We analyzed the transcription factors encoded by the differential genes in the CF-CG and F-CG comparison groups, and we identified 3907 differential transcription factors. These transcription factors mainly belong to the gene families of *bHLH* (378 genes), *MYB-related* genes (268 genes), *ERF* (255 genes), *NAC* (253 genes), *C2H2* (206 genes), *C3H* (200 genes), *WRKY* (194 genes), etc. (Figure 5F). These results indicate that these transcription factor families might play key roles in regulating the expression levels of genes related to taproot enlargement in garden ginseng.

### 2.5. qRT-PCR Validation of RNA-seq Data

**Figure 6:**
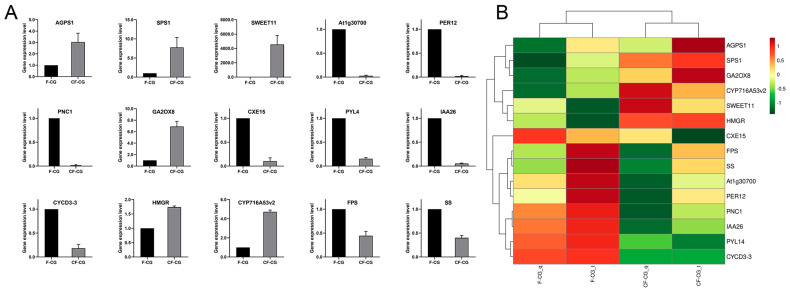
(**A**): qRT-PCR verification of differentially expressed genes in CF-CG and F-CG. (**B**): qRT-PCR and RNA sequence correlation analysis.

To validate the accuracy of our RNA-seq data, 15 genes possibly related to taproot enlargement in CF-CG, including genes encoding pathways related to starch and sucrose metabolism, phenylpropane biosynthesis, plant hormone signal transduction and ginsenoside biosynthesis, were examined using qRT-PCR (Figure 6A). A correlation analysis showed that the qRT-PCR results are consistent with the trend of RNA-seq expression levels (Figure 6B), indicating that the transcriptome data were reliable.

### 2.6. Combined Analysis of Transcriptome and Metabolome

**Figure 7:**
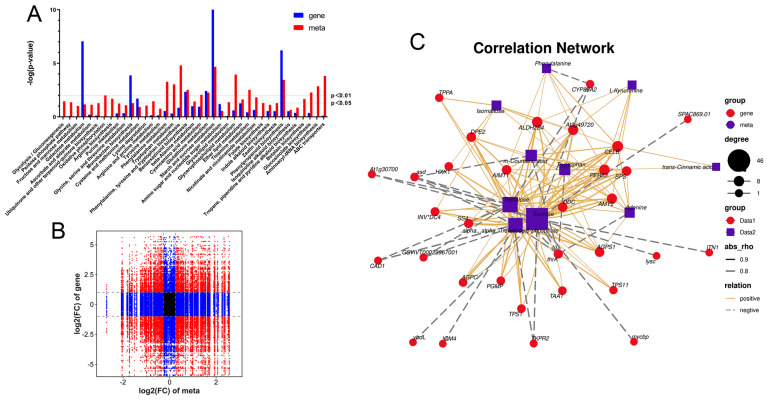
Correlation analysis of differential genes and differential metabolites in F-CG and CF-CG. (**A**): The nine-quadrant diagram shows the correlation of differential genes and differential accumulation metabolites between F-CG and CF-CG; (**B**): KEGG co-enrichment analysis of differential genes (blue column) and differential metabolites (red column); (**C**): correlation network diagram of differential genes and differential metabolites in pathways related to taproot enlargement in garden ginseng.

A co-expression analysis (CF-CG vs F-CG) was performed on the transcriptome and metabolome to explore the relationship between the DEGs and DEMs during taproot enlargement in garden ginseng. The nine-quadrant diagram illustrates the correlation between the genes and metabolites (Figure 7A). Among them, only the genes and metabolites located in the third and seventh quadrant showed consistent differential expression patterns; that is, the expression changes of the metabolites might be positively regulated by the genes. However, the expression changes of the metabolites in the first and ninth quadrants may be negatively regulated by the genes. In this study, we found that 59 DEMs in the taproot of F-CG and CF-CG may be related to 5782 DEGs. The DEMs and DEGs were found to be co-enriched in the carbohydrate metabolism pathway, amino acid metabolism pathway and secondary metabolite biosynthesis pathway in the KEGG pathway analysis, among which the starch and sucrose metabolism pathways and phenylpropane biosynthesis pathway were significantly enriched by the DEGs and DEMs (*p*-value < 0.01) (Figure 7B). Many plant hormone biosynthesis pathways are involved in the amino acid metabolism pathway and the secondary metabolite biosynthesis pathway. Interestingly, a correlation network analysis was further performed on the DEGs and DEMs (Figure 7C), which were significantly enriched in the above metabolic pathways, and it was found that α,α-trehalose 6-phosphate, sucrose and trehalose were significantly positively correlated with *ALDH2B4*, *AGPS1*, *AMY2*, *CELB*, *TPS1*, *PER63*, *DPE2*, *At3g49720*, *SPS* and *SS4* and significantly negatively correlated with *ASD*, *IQM4*, *THRA*, *CAD1*, *TKPR2* and *CYP89A2*. Tryptophan and L-kynurenine, precursors of auxin synthesis, were significantly and positively correlated with *CELB*, *PER63* and *ALDH2B4*.

## 3. Discussion

Ginseng is a perennial herb with a slow growth rate and a root phenotype affected by genetic, environmental and physiological factors [11]. At present, compared with studies on the pharmacological effects of ginseng, the number of physiological studies of ginseng is limited, and there are even less studies on root phenotypes. In this study, we evaluated the morphological differences in the taproots of CF-CG and F-CG. Compared with F-CG, the lengths of the taproots of CF-CG were not significantly different, while the roots’ thicknesses were increased by 70.5% (Figure 1C,D). The main roots of CF-CG were significantly thicker than that of F-CG, and the degree of enlargement of its main root was more significant. The levels of carbohydrates and ginsenosides were determined. At the same time, the differences between the two phenotypes of CF-CG and F-CG were analyzed using metabolome and transcriptome analyses. The results show that the starch and sucrose metabolism pathways, plant hormone signal transduction and the phenylpropane and ginsenoside biosynthesis pathways were important events for taproot enlargement in CF-CG, providing a theoretical basis for the regulation mechanism of root morphogenesis in garden ginseng.

**Figure 8:**
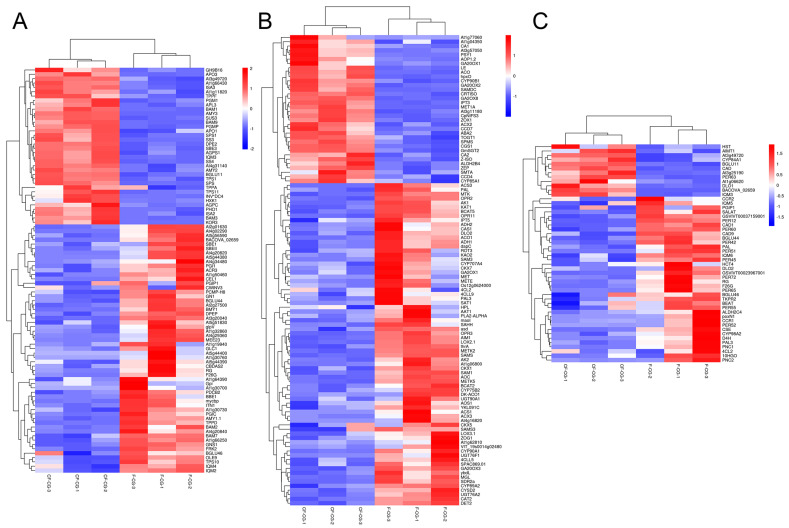
Heat map analysis of differentially expressed genes between CF-CG and F-CG. Red, up-regulated genes; blue, down-regulated genes. (**A**): Heat map analysis of differential genes in starch and sucrose metabolic pathways in F-CG and CF-CG. (**B**): Heat map analysis of differential genes in plant hormone biosynthesis and signal transduction pathways in F-CG and CF-CG. (**C**): Heat map analysis of differential genes in the phenylpropane metabolic pathways in F-CG and CF-CG.

During the growth and development of ginseng, the root is a large sink in the plant source–sink relationship, and the activity of the sink is an important criterion for measuring the swelling ability of the root. Starch is considered to be one of the main storage carbohydrates. In this study, the expression of *SS4* (a starch synthase) associated with starch biosynthesis was up-regulated in CF-CG, but, at the same time, the expressions of amyloytic enzymes, such as *AMY2* (α-amylase), *BAM2* (β-amylase) and *DPF2* (4-α-glucan transferase), were up-regulated (Figure 8A). However, compared with F-CG, the starch content in CF-CG was slightly lower, and it is possible that the decomposition rate was higher than the synthesis rate at this time, thereby promoting the decomposition of starch. Sucrose, the main product of photosynthesis, plays an extremely important role in regulating the root weight ratio. It can be transported to the roots of plants for unloading through long-distance transport, providing energy for the growth and development of the roots. We found that the contents of sucrose and fructose in the taproots of CF-CG were significantly higher than those in the taproots of F-CG (Figure 2B,C). The starch and sucrose metabolism pathways have also been demonstrated to be the key pathways for the thickening of the taproots of radish [29] and sweet potato [30]. The transcriptome data indicated that differentially expressed genes in CF-CG and F-CG were significantly enriched in the sucrose and starch metabolism (map00500) pathways, and the *SUS* gene has been found to be of great significance for the formation and development of potato tuberization [31], sweet potato rooting [18] and radish fleshy roots [29]. In our study, we also found that *SUS3* (sucrose synthase) and *SPS1* (sucrose phosphate synthase) genes were significantly up-regulated in the taproots of CF-CG, contributing to the synthesis and accumulation of sucrose (Figure 8A). Interestingly, the up-regulation of *SWEET11* (sucrose export protein) gene in the CF-CG taproots promoted the unloading of sucrose in the taproots, while the activity of *CWINV3* (cell wall acid invertase) was decreased, resulting in the higher accumulation of sucrose content in the CF-CG taproots (Figure 8A). This indicates that sucrose might play a major role in the process of the enlargement of the taproots of CF-CG.

Plant hormones have been found to be important signals for plant root development. Many studies have shown that hormone-related genes can participate in the secondary growth of cambium by regulating cell division, differentiation and expansion [32,33,34]. In this study, based on the GO and KEGG pathway annotations, the plant hormone signal transduction (map04750) pathway was the most abundant one (Figure 5C,D). We identified a total of 380 differential genes involved in plant hormone biosynthesis and signal transduction pathways, involving eight biosynthesis and metabolic pathways, namely auxin, cytokinin, abscisic acid, ethylene, brassinolide, jasmonic acid, gibberellin and salicylic acid (Figure 8B). In recent years, many studies have confirmed that hormones such as jasmonic acid, salicylic acid, brassinolide and ethylene are involved in the regulation of storage organ formation and secondary tissue development [35,36,37]. However, the expressions of related genes were mostly down-regulated in CF-CG in this study, which might have little effect on the later stages of taproot enlargement in garden ginseng.

Auxin has been shown to regulate cell proliferation and cell expansion by altering gene expression [38]. This study shows that the expression levels of the *ALDH2* and *At1g77060*(YUCCAS) genes that promote auxin biosynthesis were significantly up-regulated in CF-CG. Increasing auxin synthesis was beneficial for inducing a series of related auxin-responsive genes, which may play important roles in promoting root enlargement in CF-CG. Recently, a previous study showed that gibberellin was involved in the root growth and secondary xylem and lignin accumulation of carrots [39]. In another study, an exogenous application of gibberellin GA3 could significantly down-regulate genes related to carbohydrate metabolism and starch biosynthesis. This, in turn, promoted the lignification of storage roots, inhibited the expansion of plant roots and significantly reduced the number and diameter of storage roots [40]. However, compared with F-CG, this study found that the up-regulated expression of GA synthesis genes (*GA20ox1* and *GA20ox2*) and the down-regulated expression of GA lyase genes (*GA20ox3* and *GA2ox1*) in CF-CG were conducive to the accumulation of GA content, which is consistent with the study conducted by Hong [19] et al., who found that the exogenous application of GA promoted the secondary growth of roots in ginseng. More interestingly, ABA generally reduces cell growth and division [41]. However, compared with F-CG, the expression of key genes involved in the ABA synthesis pathway, such as *ZEP, CCD4* and *ABA2*, were significantly up-regulated in the CF-CG taproots. Furthermore, *CYP707A*, an important factor promoting ABA degradation [42,43], was significantly down-regulated in the CF-CG taproots, which was conducive to the accumulation of ABA content, indicating that ABA had a positive regulatory role in the enlargement of the CF-CG taproots. In summary, compared with F-CG, the synthesis-related genes of auxin, gibberellin and abscisic acid were significantly up-regulated in CF-CG, which might play important active roles in promoting the enlargement of main root in CF-CG. However, the root development of plants is not the result of a single regulation by a certain hormone, but the interaction of multiple hormones that work together to regulate the enlargement of garden ginseng taproots; this needs to be further studied.

Ginsenosides, the main medicinal components of ginseng, belong to the group of triterpenoids, which play important roles in plant defense and the growth and development of ginseng [44]. Studies have shown that, when different concentrations of ginsenosides Re and Rb1 are exogenously applied, Rb1 inhibits the adventitious root branching of ginseng, while Re can promote the adventitious root branching of ginseng at an appropriate concentration [22,45]. Our study found that the contents of nine types of ginsenosides and total saponins in the taproots of CF-CG were higher than those in the taproots of F-CG (Figure 3), especially the ginsenosides Rb1 and Rb2 (Figure 3A,B). The accumulation of ginsenosides Rb1 and Rb2 may be related to the enlargement of the taproots of garden ginseng. In addition, compared with F-CG, in the phenylpropane metabolic pathway (map00940), the genes related to the lignin synthesis pathway in the taproots of CF-CG, such as *PAL*, *CYP98A2*, *4CL2*, *CCR* and *CAD1*, were found to be significantly down-regulated (Figure 8C). There was a correlation between changes in the transcription level of lignin biosynthesis genes and lignin accumulation [46], which induced the transcription level of lignin biosynthesis genes, promoted the lignification of roots, and then reduced the formation and enlargement of storage roots [30,40]. From this point of view, the biosynthesis of lignin was inhibited, thereby reducing the degree of lignification during the process of the enlargement of the taproots of CF-CG.

During the vegetative growth of ginseng, cells were stimulated by environmental factors, such as light, temperature and soil. They received and transmitted various signaling molecules; genes related to starch and sucrose metabolism were up-regulated and genes related to lignin synthesis were down-regulated in CF-CG. Additionally, there were also some regulatory factors, such as *bHLH*, *MYB_related*, *ERF* and *NAC*, that participated in the differentiation, division and expansion of the secondary xylem and phloem of garden ginseng, thus promoting the enlargement of the taproots of garden ginseng. Studies have shown that sugar signaling molecules can interact with plant hormones to regulate plant growth and development [47]. α,α-trehalose 6-phosphate (T6P) was the main differential metabolite and an important signaling metabolite in the taproots of CF-CG. It is the precursor of trehalose synthesis and the intermediate product between sucrose and trehalose, playing an important role in the growth and development of plants. Studies have shown that auxin is a key factor mediating the action of T6P, which acts on *TAR2*, a key auxin synthesis gene, to trigger the storage and development of pea seeds [48]. A similar regulation was found in this study; *ALDH2* was a key gene in auxin synthesis, and it had a significant positive correlation with T6P (Figure 7C). It was speculated that T6P acted on *ALDH2* to regulate auxin synthesis, promoted cell division and expansion and, thus, caused the enlargement of the taproots of the garden ginseng.

## 4. Materials and Methods

### 4.1. Plant Material

The roots of garden ginseng plants were used as experimental material. At the beginning of September 2020, samples of cutting forest ginseng (CF-CG) and farmland ginseng (F-CG) were collected in the cultivation bases of Fusong County (42°33′ N, 127°27′ E) and Liuhe County (40°88′ N, 125°7′ E) in Jilin Province of China, respectively. CF-CG and F-CG were grown under natural conditions. These plants were excavated from the soil and washed with ultrapure water, and all taproot tissues of the three plants were mixed as a biological replicate and immediately snap-frozen with liquid nitrogen. Three biological replicates were collected from CF-CG and F-CG for transcriptome sequencing; six biological replicates were collected for metabolome sequencing. The remaining ginseng root samples were separated into taproots, rhizomes and lateral roots, and then they were dried at 45 °C to a constant weight for determining the contents of ginsenoside and carbohydrates. The differences between the roots of CF-CG and F-CG are shown in Figure 1A,B.

### 4.2. Determination of Carbohydrate Contents in Ginseng

The dried powder of the taproot tissues was taken and weighed three times, weighing 1.000 g each time. After adding distilled water, ginseng polysaccharide was extracted using a microwave (MARS6, Matthews, NC, USA) (microwave power 600 w, solid–liquid ratio 1:30, extraction time 6 min, extraction temperature 70 °C) and centrifugal filtration (4500 r/min, 10 min), and it was transferred to a volumetric flask with a volume of 25 mL, followed by the addition of anhydrous ethanol four times. After standing at 4 °C for 12 h, centrifugal filtration was carried out (4500 r/min, 10 min), and the supernatant was removed to obtain precipitate. The precipitate was evaporated to dryness in a water bath, reconstituted with distilled water and made up to a volume of 100 mL in a volumetric flask. Then, 1 mL of ginseng extract was mixed with 1 mL of 5% phenol and 5 mL of concentrated sulfuric acid, and this sample was put in a water bath at 100 °C and allowed to react for 20 min. After the reaction time was reached, it was quickly cooled to room temperature with an ice-water bath, and the absorbance of the samples was measured with a spectrophotometer (detection wavelength 490 nm).

An aqueous solution containing a D-glucose standard substance was prepared and diluted to the appropriate concentration to establish a standard curve. The standard curve was constructed by plotting the absorbance and the concentration of D-glucose. To calculate the total polysaccharide content in the taproot tissue of the ginseng, the standard regression equation used was total polysaccharide: Y = 16506X – 0.0178 (R^2^ = 0.9996). The dried powder of the taproot samples was weighed three times, weighing 1.000 g each time, and the contents of glucose, sucrose, fructose and starch in the taproots of CF-CG and F-CG were determined by using a glucose assay kit (F006-1-1), a sucrose measurement kit (A099-2-1), a fructose assay kit (A085-1-1) and a starch content kit (A148-1-1) (Nanjing Jiancheng Bioengineering Institute, Nanjing, China).

### 4.3. Determination of Monomeric Saponins and Total Saponins in Ginseng

The dried powder obtained from each part of the samples was weighed three times, weighing 1.000 g each time. After being immersed in methanol overnight, ginsenosides were extracted using ultrasound (extraction conditions: ultrasonic frequency 40 kHz, extraction temperature 30 °C, extraction time 45 min and solid-to-liquid ratio 1:30). The filtered solution was evaporated in an evaporating dish, reconstituted with methanol and transferred to a 10 mL volumetric flask; the volume was adjusted to 10 mL. It was shaken well and filtered through a 0.22 μm filter for an HPLC analysis.

An analysis was performed using an Agilent 1260 generation II HPLC system (Agilent, CA, USA) equipped with an autosampler, a four-stage gradient pump and an Agilent SB-C18 column (4.6 mm × 250 mm, 5 μm) and UV detector. The gradient elution system includes A (100% water) and C (100% acetonitrile) for gradient elution and the following gradient program: 0–40 min: 79% A; 40–42 min, 74% A; 42–46 min, 68% A; 46–72 min, 60% A; 72–89 min, 40% A; 89–92 min, 35% A; 92–98 min, 15% A; 98–102 min, 0% A; and 102–106 min, 81% A. The injection volume was 10 μL, the flow rate was 0.8 mL/min, the system temperature was 27 °C, and the detection wavelength was set to 203 nm.

An aqueous methanol solution containing ginsenoside Rg1, ginsenoside Re, ginsenoside Rf, ginsenoside Rb1, ginsenoside Rc, ginsenoside Rg2, ginsenoside Rb2, ginsenoside Rb3 and ginsenoside Rd was prepared and diluted to an appropriate concentration for the establishment of a standard curve. Five concentrations of nine monomeric saponins were detected, and the standard curve was constructed by plotting the peak area versus the concentration of each analyte. The standard regression equations for ginsenoside Rg1, ginsenoside Re, ginsenoside Rf, ginsenoside Rb1, ginsenoside Rc, ginsenoside Rg2, ginsenoside Rb2, ginsenoside Rb3 and ginsenoside Rd were as follows: Rg1: Y = 471.1X + 7.3252 (R^2^ = 0.9999); Re: Y = 385.02X + 5.7274 (R^2^ = 0.9997); Rf: Y = 357.2X + 0.9383 (R^2^ = 0.9991); Rb1: Y = 276.14X + 5.6388 (R^2^ = 1); Rc: Y =239.24X + 5.5644 (R^2^ = 0.9999); Rg2: Y = 450.48X + 2.9523 (R^2^ = 0.9998); Rb2: Y = 269.98X + 3.0672 (R^2^ = 0.9999); Rb3: Y = 269.55X + 0.7054 (R^2^ = 0.9994); and Rd: Y = 286.91X + 0.9281 (R^2^ = 0.9992). The peak areas of the nine ginsenoside monomer saponins were measured, and the contents of the nine ginsenoside monomer saponins were calculated according to the above standard regression equations.

The extracted ginsenoside alcohol solution was placed in a test tube with a mouthpiece stopper, and the methanol was evaporated to dryness in a water bath at 60 °C. Then, 0.5 mL of an 8% vanillin ethanol solution and 5 ml of a 72% concentrated sulfuric acid solution were added, shaken and placed in a water bath at 45 °C. After heating for 10 min, the solution was immediately cooled to room temperature in an ice-water bath and shaken well, and the absorbance was measured with a spectrophotometer (detection wavelength: 544 nm). A methanol solution of a ginsenoside Re standard substance was prepared and diluted to an appropriate concentration to establish a standard curve. The standard curve was constructed by plotting the absorbance and the concentration of ginsenoside Re. To calculate the total saponin content in the taproot tissue of the ginseng, the standard regression equation used was ginsenoside Re: Y = 4.9408X − 0.0333 (R^2^ = 0.9995).

### 4.4. Metabolome Analysis

The samples of the ginseng taproots, including those of CF-CG and F-CG (*n* = 6), that had been stored at −80 °C, were thawed slowly on ice, and 120 µL of a prechilled 50% methanol buffer was used to extract the metabolites from 20 µL of each sample. The metabolite mixture was vortexed for 1 min, incubated at 20–25 °C for 10 min and stored at −20 °C overnight. The mixture was centrifuged at 4000× *g* for 20 min, and the supernatant was transferred to a 96-well plate. The samples were stored at −80 °C before an LC-MS analysis. Mixed quality control (QC) samples were prepared by combining 10 μL of each extraction mixture.

All samples were analyzed using a Triple TOF 5600 Plus high-resolution tandem mass spectrometer (SCIEX, MA, USA) with both positive and negative ion modes. Chromatographic separation was performed using an Ultra-Performance Liquid Chromatography (UPLC) system (SCIEX, MA, USA). An ACQUITY UPLC T3 column (100 mm × 2.1 mm, 1.8 µm, Waters, MA, USA) was used for reversed-phase separation. It was introduced for the separation of metabolites, and the mobile phase consisted of solvent A (water, 0.1% formic acid) and solvent B (acetonitrile, 0.1% formic acid). The gradient elution conditions were as follows: a flow rate of 0.4 mL/min: 5% solvent B for 0–0.5 min; 5–100% solvent B for 0.5–7 min; 100% solvent B for 7–8 min; 100–5% solvent B for 8–8.1 min; and 5% solvent B for 8.1–10 min. The column temperature was maintained at 35 °C.

The Triple TOF 5600 Plus system was used to detect the metabolites eluted from the column. The curtain gas pressure was set to 30 PSI, and the ion source gas1 and gas2 pressure was set to 60 PSI. The interface heater temperature was 650 °C. For the positive-ion mode, the ion spray floating voltage was set to 5 kV, and for the negative-ion mode, it was set to −4.5 kV. The MS data were acquired in the IDA mode. The TOF mass range was 60–1200 Da. The total cycle time was fixed at 0.56 s. Four time bins were summed for each scan at a pulse frequency of 11 kHz by monitoring the 40 GHz multichannel TDC detector with four-anode/channel detection. Dynamic exclusion was set for 4 s. During the entire acquisition period, the mass accuracy was calibrated every 20 samples. Furthermore, a QC sample was analyzed every 10 samples to evaluate the stability of the LC-MS.

The acquired LC-MS data were preprocessed using XCMS software. Raw data files were converted into m/z XML format and then processed using the XCMS, CAMERA and metaX toolbox included in R software. Each ion was identified by the comprehensive information of retention time and m/z. The intensity of each peak was recorded, and a three-dimensional matrix containing arbitrarily assigned peak indices was developed (retention time-m/z pairs). Then, the information was matched to the in-house and public databases. The open-access databases KEGG and HMDB were used to annotate the metabolites by matching the exact molecular mass data (m/z) to those from the database within a threshold of 10 ppm. The peak intensity data were further preprocessed using metaX. The features that were detected in <50% of the QC samples or 80% of the test samples were removed, and the values for the missing peaks were extrapolated with the k-nearest neighbor algorithm to further improve the data quality. In addition, the relative standard deviations of the metabolic features were calculated across all QC samples, and those with standard deviations >30% were removed. The group datasets were normalized before the analysis was performed. Data normalization was performed on all samples using the probabilistic quotient normalization algorithm. Then, QC-robust spline batch correction was performed using the QC samples. The *p* value analyzed using Student *t*-tests and then adjusted for multiple tests using an FDR (Benjamini–Hochberg) was used for the different metabolite selection. We also conducted a supervised PLS-DA using metaX to identify variables, and a discriminant profiling statistical method to identify more specific differences between the groups. The VIP cut-off value of 1.0 was set to select important features.

### 4.5. Transcriptome Analysis

The total RNA was extracted according to the instruction manual of the Trizol reagent (Invitrogen, CA, USA). The total RNA quantity and purity were analyzed using the Bioanalyzer 2100 and an RNA 1000 Nano Lab Chip Kit (Agilent, CA, USA) with RIN number >7.0. Poly(A) RNA was purified from total RNA (5 ug) using poly—Toligo-attached magnetic beads with two rounds of purification. Following purification, the mRNA was fragmented into small pieces using divalent cations under an elevated temperature. Then, the cleaved RNA fragments were reverse transcribed to create the final cDNA library in accordance with the protocol for the mRNA-Seq. sample preparation kit (Illumina, CA, USA), and the average insert size of the paired-end libraries was 300 bp (±50 bp). Then, we performed paired-end sequencing on an Illumina Novaseq™ 6000 (LC Sceiences, TX, USA) following the vendor’s recommended protocol.

Firstly, Cut adapt and perl scripts were used in house to remove the reads that contained adaptor contamination, low-quality bases and undetermined bases. Then, sequence quality was verified using Fast QC (http://www.bioinformatics.babraham.ac.uk/projects/fastqc/ accessed on 11 January 2021), including the Q20, Q30 and GC content of the clean data. All downstream analyses were based on high-quality clean data. The de novo assembly of the transcriptome was performed with Trinity 2.4.0. Trinity groups transcripts into clusters based on shared sequence content. Such a transcript cluster is very loosely referred to as a ‘gene’. The longest transcript in the cluster was chosen as the ‘gene’ sequence (also known as a unigene). All assembled unigenes were aligned against the non-redundant (NR) protein database (http://www.ncbi.nlm.nih.gov/ accessed on 11 January 2021) and the Gene Ontology (GO) (http://www.geneontology.org/ accessed on 11 January 2021), SwissProt (http://www.expasy.ch/sprot/ accessed on 11 January 2021), Kyoto Encyclopedia of Genes and Genomes (KEGG) (http://www.genome.jp/kegg/ accessed on 11 January 2021) and eggNOG (http://eggnogdb.embl.de/ accessed on 11 January 2021) databases using DIAMOND with an E value threshold of <0.00001.

### 4.6. qRT-PCR

Salmon was used to determine the expression levels of the unigenes by calculating TPM. The differentially expressed unigenes were selected with log2(fold change) > 1 or log2(fold change) < −1 and with statistical significance (*p* value < 0.05) using R package edger. To validate the RNA-seq. data, 15 DEGs were selected for qRT-PCR (Table S1). The ginseng root gene *GAPDH* served as the reference gene. FC was estimated in terms of threshold cycles according to the 2^−ΔCT^ method. Three biological replicates and three technical replicates were assessed.

### 4.7. Statistical Analysis

The original data were compiled using MS Excel 2019 software (Microsoft, Redmond, WA, USA), and SPSS 19.0 was used for data analyses (IBM SPSS 27, Armonk, NY, USA). A bioinformatic analysis was carried out and graphics were generated using OmicStudio tools (https://www.omicstudio.cn/tool), and GraphPad Software (version 7.0, GraphPad Software Inc., MA, USA) was used to draw the graphics to better present the experimental results.

## 5. Conclusions

Our research shows that the morphology of garden ginseng taproots is different in different environments. The taproots of CF-CG planted in deforested areas were enlarged, and the root thickness was increased by 70.5% compared with that of F-CG. More metabolites, such as sucrose, fructose, ginsenoside Rb1, Rb2 and total saponins, were accumulated in the CF-CG taproots. A total of 9,960 DEGs were identified using a transcriptome analysis, and the KEGG analysis revealed significant changes in the starch and sucrose metabolism, phenylpropane biosynthesis, and plant hormone signal transduction pathways. According to a metabolome analysis, the contents of metabolites were highly correlated with gene expressions, and the synthesis of carbohydrates, lignin, plant hormones and ginsenosides, as well as the related genes, played important roles in the enlargement of the taproots of the CF-CG. In addition, we speculate that T6P, as a sugar signaling molecule, could act on the auxin synthesis gene *ALDH2* to promote the synthesis of auxin in order to regulate taproot enlargement, which needs further investigation. In summary, our study lays the foundation for clarifying the regulatory mechanism of taproot enlargement in garden ginseng, with a view to providing new insights into the morphogenesis of garden ginseng roots.

## Figures and Tables

**Table 1:** The RNA-seq data for these 6 samples.

Sample	Raw_Reads	Raw_Bases	Valid_Reads	Valid_Bases	Valid%	Q20%	Q30%	GC%
CF-CG_1	58,387,112	8.76G	57,132,936	7.99G	97.85	98.06	93.91	43.49
CF-CG_2	57,994,892	8.70G	56,941,662	7.96G	98.18	97.97	93.69	43.46
CF-CG_3	34,002,510	5.10G	33,173,734	4.64G	97.56	98.01	93.54	43.59
F-CG_1	58,074,704	8.71G	57,342,028	8.02G	98.74	97.96	93.61	43.64
F-CG_2	58,443,348	8.77G	57,543,876	8.04G	98.46	97.97	93.65	43.48
F-CG_3	54,182,540	8.13G	53,095,988	7.42G	97.99	97.97	93.68	44.06

**Table 2:** Summary of assembly results of taproot in garden ginseng.

Index	All	GC%	Min Length	Median Length	Max Length	Total Assembled Bases	N50
Transcript	241,332	39.46	201	679.00	15,897	229,477,537	1430
Gene	65,913	39.42	201	512	15,897	57,009,225	1476

**Table 3:** Summary of function annotation of taproot in garden ginseng.

DB	All	GO	KEGG	Pfam	Swissprot	eggNOG	NR
Num	65,913	30,113	23,104	25,738	24,622	33,498	34,141
Ratio (%)	100	45.69	35.05	39.05	37.36	50.82	51.8

## Data Availability

The datasets for this study are available in this manuscript and the Supplementary Materials.

## Supplementary Materials

The following supporting information can be downloaded at: https://www.mdpi.com/article/10.3390/ijms24065590/s1.
